# Fake bus stops for persons with dementia? On truth and benevolent lies in public health

**DOI:** 10.1186/s13584-019-0301-0

**Published:** 2019-03-07

**Authors:** Pauline Lorey

**Affiliations:** Institute of History and Ethics in Medicine, Friedrich-Alexander-University, Glückstraße 10, 91054 Erlangen, Germany

**Keywords:** Dementia, Wandering persons with dementia, Fake bus stops, Truth, Deception, Benevolent lies, White lies, Public health, Medical ethics, Bioethics

## Abstract

Fake bus stops are one strategy to keep persons with dementia (PwD) from wandering. By setting up authentic looking shelters and benches in hallways or gardens, nursing homes create the illusion of bus stops, for the purpose of preventing wandering PwD from leaving the facility and getting lost. By attracting wandering PwD to sit down and wait for the bus, they can be supervised more easily by caregivers. However, concerns are expressed that the use of fake bus stops could cause more harm than good for PwD, due to their deceptive nature and the potential stigmatisation of individuals seated at a fake bus stop. This article discusses the ethical aspects of using fake bus stops and outlines considerations prior to setting up fake bus stops in nursing homes in keeping with good clinical practice in dementia care. Moreover, the article assesses whether or not fake bus stops can be ethically justifiable, and if so, how they can be ethically justified and implemented in Israeli and other facilities for PwD.

## Introduction

As the number of persons with dementia (PwD) rises, strategies are being developed to address their needs [1]. One serious problem associated with dementia is wandering. Wandering can be stressful for caregivers and healthcare institutions.

Fake bus stops are one attempt to prevent PwD from wandering within, as well as, out of, nursing homes. These stops are sometimes outfitted with information boards, fake timetables, and actual bus stop signs, but a bus is never actually going to arrive. Benches or booths simply resemble real bus stops and are erected in the corridors of nursing homes or in the facility’s garden.

Fake bus stops were introduced in Germany over 10 years ago, with the intention of reducing wandering among older individuals. However, their has been controversial [2]. This debate has become an international discourse in both the media and scientific publications. [3–6] .

To illustrate the seemingly absurd use of these fake bus stops, some refer to famous plays like “Waiting for Godot” (in which two characters are waiting for the arrival of a person called Godot, who never arrives) [7, 8] or the film “The Truman Show” (in which the main character, Truman Burbanks, unwittingly participates in a television show built around his simulated life) [9, 10]. The relevant and controversial nature of this topic was further illustrated by the animated short film by Australian director Adam Elliot, named “Harvie Krumpet”, which depicted a fake bus stop. In two short scenes, the main character, Harvie Krumpet, who is diagnosed with Alzheimer’s disease, sits at a fake bus stop waiting for a bus that never arrives. After its premiere, the short film received international attention and even won an Academy Award for Best Animated Short Film in 2004 [9, 10].

Fake bus stops may restrict PwD’s autonomy and can lead to their stigmatisation. As a direct result of PwDs’ cognitive deficits and inability to understand, one can argue that they are being taken advantage of in this fake bus stop illusion. Debunking this deception may cause distress to a person with dementia. Hence, this raises the question whether building fake bus stops thoughtlessly could cause more harm than benefit.

Despite the controversy that fake bus stops may cause, it has alos been also argued that the use of fake bus stops in nursing homes for older persons may help improve the care of PwD by giving them a place to go when they are inclined to wander [4]. There are those who claim that in selected cases, fake bus stops can provide a safe retreat for PwD, which prevents PwD from walking out onto the streets.

This article discusses the ethical issues of using fake bus stops and deliberates on good clinical practice in dementia care. Are fake bus stops an appropriate strategy for Israeli nursing homes to keep PwD from wandering? If so, does their potential benefit outweigh the potential harm they may cause? And finally, what ethical considerations should Israeli practitioners and nursing homes consider before building fake bus stops?

## Persons with dementia, deception and public health ethics

### The issue of wandering PwD in Israel and Germany

In 2015, a study estimated the percentage of people over the age of 65 to be 11% in Israel [11], a low percentage compared to other developed nations (e.g. 21,1% in Germany in 2016 [12]). The number of older persons is projected to double within the next 20 years [11]. The increased age of society and of age-related illnesses, as well as the financing of the public health system are expected to cause many challenges in Israel. Three potential challenges in Israel are of particular long-term concern: the lack of universal coverage, the multiplicity of authorities charged with overseeing and managing the sector, and the lack of preparation for the changing demographics of the future [13]. Approximately 2% of the older population lived in nursing homes or other care facilities in Israel in 2015 [11]. For older PwD living in care facilities, the implementation of fake bus stops could provide an opportunity to cope with behavioral and psychological symptoms such as wandering. This is further discussed below.

The symptoms of dementia can be grouped into two categories: the first category, cognitive functions, such as impairment of memory, communication, ability to focus, reasoning, and visual perception [14]. The second category includes behavioral and psychological symptoms of dementia (BPSD), such as depression, anxiety, wandering and agitation, repetitive questioning, and sexual disinhibition [15]. BPSD often reflect great distress experienced by PwD [16] and causes great distress in their caregivers. Thoughtful and attentive caregivers are essential for good dementia care. Caring for PwD often requires the caregivers to supervise and ensure that they do not leave the facilities. Frequency and disruptiveness of agitated behavior in PwD are correlated with a higher level of perceived burden amongst caregivers and therefore affect the caregivers’ well-being and ability to perform their work effectively [17]]. Moreover, these symptoms can cause premature hospitalisation of PwD and thereby generate higher costs of care [15, 18, 19].

Wandering, an aberrant motor behavior, is one of the most common behavioral symptoms PwD display. Wandering is challenging for caregivers but is not dangerous in itself for PwD. However, the urge to wander in combination with the PwD’s loss of orientation can lead to dangerous situations, e.g. PwD may get lost and be unable to find his or her way home. In addition, traffic poses a major risk for a person with cognitive deficits.

In response to these risks, a number of nursing homes in Germany have set up fake bus stops to prevent PwD from wandering. In the following, the benefits and risks of this intervention will be discussed.

### Design and purpose of fake bus stops

PwD often manifest agitation [17], which can be expressed as increased level of excitement, anxiety, or in abnormal motor behavior. Due to a lack of appropriate medical and non-medical interventions, agitation is still a problem in dementia care [20]. Agitated PwD may leave their nursing homes and wander around aimlessly. In an effort to reduce the risk of PwD getting lost, nursing homes have been searching for methods to reduce wandering behavior.

To counter these behavioral and psychological symptoms, nursing homes tend to prescribe antipsychotic medication to PwD. The excessive use of antipsychotic medications is problematic due to their potential side effects. These side effects range from sedation, to parkinsonism, increased risk of infections, and increased mortality [21]. The use of antipsychotic medication was associated with a shortage of occupational therapists and social workers in a study conducted in Israel [22]. Due to a a lack of manpower, infrastructure and funding, there is a great need for creative solutions for alternatives to antipsychotic medication.

Fake bus stops could offer an alternative solution to many of the same problems that anti-psychotics attempt to address. Fake bus stops vary in design and form. Some nursing homes put up complete bus stop shelters, which look exactly like the real ones on the street. Often they offer seating such as benches or individual seats.

To make bus stops appear more realistic, some nursing homes decorate them with information boards, fake timetables, or actual bus stop signs. The displayed timetables may show real bus schedules, fictional bus schedules, or the schedule of the daily nursing home routine. There are differences in fake bus stop designs and deception. Arguably, the more authentic a fake bus stop looks, the more deceptive it is. Fake timetables, for example, are more deceptive than daily schedule flyers, because fake timetables are more authentic. Another deceptive feature of fake bus stops is the eye-catching yellow and green bus stop sign with a green “H” for “Haltestelle” (German for bus stop). This is the standardised bus stop sign across Germany. Similar to these “H” signs are the yellow bus stop signs used in Israel, which display the name of the stop, bus line numbers, and their destination. The purpose of displaying standardised bus stop signs is quick and easy recognition of bus stops location. The purpose of the fake bus stops is to deceive, therefore, in this article, we will focus on authentically designed fake bus stops.

In the following section, we will discuss the risks and benefits that are involved with the use of fake bus stops as an intervention to prevent PwD from wandering.

### Truth, lies, and deception in dementia care

Lying is a largely controversial and commonly discussed topic in the context of dementia. Previous work reported that more than 90% of carers occasionally lie to the PwD they care for [23–25]. Yet, there is a range of perspectives when it comes to the definition of a truth and a lie, demonstrating each of the terms’ contested character.

Lying is defined as either applying a falsification or as purposely altering facts in a way to mislead a person [26]. Kant held a deontological philosophical position that forbids both lies and deception under any circumstance. According to Kant, being truthful in all declarations is a universal maxim [27].

In contrast, a ultilitarian perspective views the morality of an action as determined by its consequences [28]. This suggests that lying can be justified if it is in the person‘s (to whom the lie is told) best interests [24], or in situations where someone is at risk of injury or harm [29]. In fact, recent studies that examined the perspectives of caregiver staff [24], clinical psychologists [30], and PwDs themselves [31] found that while all these actors generally expressed reservations towards lying, they were also inclined to deem it acceptable under specific circumstances. Across these studies, such specific circumstances included the person’s best interest, mainly in an effort to maintain the PwDs‘and others‘safety and prevent or reduce the PwD‘s distress. However, others rejected lying as a solution, condemning it as “away out” [32], or as an expression of “poverty of imagination” [33]. Individuals also referred to the damage of trust in relationships that lying causes between the PwD, his family and friends or between the PwD and the caregiver [34]. There are those that rejected lying and deemed it unethical and not justified even if it reduced suffering [35].

If we apply the two contrasting perspectives of utilitarianism and deontologicalism to the bus stop example, it is evident that from a Kantian perspective, fake bus stops are unacceptable because they convey deception. In her discussion of the deontological perspective, J Graf-Wäspe [9] explained that the morality of an act can be determined according to four criteria, 1. The act itself needs to be morally good or at least neutral, 2. It must not intend a negative effect, 3. The positive effect must result from the act and not from the negative effect, and 4. The positive effect must be desirable in order to compensate for the negative effect. She concluded that the installation of fake bus stops is unacceptable from the deontological perspective because the intention of deception is in itself a bad act and a negative effect of deception is intended [9].

From a utilitarian perspective, the installation of fake bus stops is deemed acceptable if driven by the assumption that it will contribute to PwD‘s physical and psychological comfort and enhance the well-being of PwD, regardless of their deceptive nature.

As part of a study on the use of truth and lies in dementia care, the Mental Health Foundation worked with five terms: whole-truth telling, looking for an alternative meaning, distraction, going along with lying, and lying [36]. They recommended, that “one should always start from a point as close to whole-truth-telling as possible – always underpinned by respect and kindness towards the person with dementia – and if this is causing unnecessary distress, move on to a response that might include an untruth.” (p. 4). The Mental Health Foundation also highlights that practice should be reinforced by respect and kindness through the use of benevolent intention. This statement expresses benevolent intent and is reminiscent of the utilitarian perspective of acting in the person’s best interest.

A fake bus stop can be classified as a deception, since people without dementia recognise it as fake, whilst PwD might falsely believe it to be real. When individuals, standing at the fake bus stop, say that: “the bus will arrive in a few minutes” the fake bus stop becomes a lie. It is deceptive to simply omit the truth (that it is not a bus stop and no bus will come). Therefore, one can argue that the ethical validity of fake bus stops depends on how people sitting at a fake bus stop are treated. Nursing staff and caregivers should consider the vulnerability of PwD. According to A Kirtley and T Williamson [36] the intention should be to stay as close as possible to the truth and therefore it is not acceptable to worsen their situation by intensifying the deception such that it becomes a lie, regardless of one’s intention.

## The risk of stigmatisation of persons with dementia

In addition to ethical dilemmas, there are also a range of risks involved in fake bus stops. Some risks were mentioned earlier, such as: the negative impact on interpersonal relationships, damage of trust to relationship of the PwDs with their caregivers, families or friends when a lie is discovered as such [34] and potential stigmatisation of PwD. Fake bus stops are built to offer an alternate reality to PwD. To achieve this, nursing homes use authentic looking elements to enhance the experience as a real one. Nursing home visitors, in contrast to PwD understand the deceptive nature of fake bus stops and are likely to conclude that individuals who sit at these stops suffer from dementia. Individuals not suffering from dementia may pity PwD, or even mock and deride them for not understanding the deception and instead choosing to sit at a fake bus stop. Setting up fake bus stops in corridors or publicly accessible places in nursing homes puts PwD, who are already more vulnerable due to their impaired cognition, at a higher risk of being stigmatised. Thus, when setting up fake bus stops, nursing homes should always consider this risk of choose a more private place over a crowded and public one to erect fake bus stops. Thus instead of building fake bus stops in corridors, foyers, or entrances of nursing homes, they should instead be built in common and recreational rooms. While it is not possible to dissuade people from judging those with dementia, it is the caregivers’ obligation to ensure the well-being of PwD and to minimize the exposure of their cognitive deficits to others.

Deceiving PwD at fake bus stops can infringe upon their right to autonomy. As their loss of cognitive functions progresses in PwD, they may lose the ability to differentiate between a truth and a lie. To debunk a lie, the person who is lied to must know the real facts behind the false statements. Mental orientation in time, space, and recognition of people are fundamental to this capacity. These abilities usually begin to disappear as cognitive symptoms of dementia intensify. It is therefore more controversial to lie to PwD than to people with unimpaired cognition since PwD lose the ability to detect when they are being deceived.

Without the cognitive ability to realise whether a situation is deceptive or false, PwD cannot choose to leave the situation. When confronted with fake bus stops, PwD are not able to choose the truth over the lie because they do not comprehend the deceptive character of fake bus stops. Therefore, PwD are deprived of their choice to leave fake bus stops, which means that the autonomy of PwD is diminished – both by dementia itself, and by fake bus stops.

Another argument against fake bus stops is that it may worsen the PwD’s confusion. As PwD lose their cognitive functions, they can also lose their comprehension of ordinary situations. In losing their ability to comprehend situations, they occasionally do not understand that it is not possible for a bus to arrive inside a nursing home, or they integrate the bus stops into their own self-created reality. PwD sometimes experience and interpret situations and surroundings differently, which can, in some instances, be difficult to understand for people without dementia. The Mental Health Foundation alludes to this confusion and argues that fake bus stops could even intensify it [36]. Furthermore, depending on their cognitive state, PwD are sometimes able to debunk a deception. By understanding that they were deceived, it may harm their well-being. Consequently, there is a risk that fake bus stops can also worsen PwDs’ well-being. As mentioned earlier, fake bus stops may not only harm PwD, but also affect the relationship between the caregiver and the PwD. As Maartje Schermer argues: “Lying to or deceiving PwDs can severely damage trust and so undermine the care relationship” [37]. M Schermer [37] refers not only to the damaged trust between caregivers and PwD that result from lies, but also to the damaged trust in an entire practice of care. A care relationship is based on mutual trust. By employing deception as a common practice, nurses and practitioners could forfeit the trust of the general public. When people observe a nurse or physician being dishonest to PwDs, it may cause distrust in healthcare professionals. Lying to or deceiving PwD, then, might not only harm PwDs but, in the long term, may also harm the perception individuals have of health professionals as well.

Physical and psychological comfort is a subjective measure, which individuals with dementia often cannot express verbally. For this reason, thoughtful caregivers who are familiar with the person with dementia, and are able to interpret the PwD’s behavioral symptoms, play an important role in providing insight into the effect of fake bus stops [20, 38, 39]. Caregivers, who are able to recognise behavioral signals in a PwD, are essential in dementia care. Caregivers who understand the PwD’s behaviour can provide care designed specifically for the needs of the individual with dementia. Training and support of care home staff can also result in a decrease of use of antipsychotic medication without worsening behavioral symptoms [38].

This emphasises the need for training and guidelines for staff about how to communicate with PwDs. Lying, although it should be avoided, can be acceptable under certain circumstances and care staff should be trained to lie in a respectful and ethical manner, while acting in the PwD’s best interest [30, 31]. This argument is reminiscent of the Declaration of Geneva, which physicians, worldwide, regard as their guiding principle. One of its first sentences is: “the health and well-being of my patient will be my first consideration” [40], which supports a consequentialist view by putting the best outcome over veracity. Only in the sentence afterwards is the indication given to autonomy and dignity of man. Literally, it states: “I will respect the autonomy and dignity of my patient”. By putting well-being in such an outstanding position (“first consideration”), one could conclude that well-being is of greater value than autonomy and dignity of man. Surmising that fake bus stops do enhance the well-being of PwD, one could conclude that they are a permissible method for PwD, because it seems more important to enhance well-being of the individual than to respect the person’s autonomy in the Declaration of Geneva.

## Alternatives to fake bus stops?

It has been argued that while lying or “therapeutic lying” [30, 41] to PwD is accepted under certain circumstances, it should always be treated as a last resort [42].

Several non-pharmacological methods are worth noting as presenting alternatives to fake bus stops. One is a preventive solution called “subjective barriers”, which are barriers that are perceived and managed differently by each person [43]. An example of such a subjective barrier is a special door, which is too heavy for older adults to open. Another strategy is to disguise the ward exits, so PwD do not recognise them as such, thus presenting an almost insurmountable barrier for them to exit through. At the same time, a person without any cognitive impairment can identify the exit as such and overcome the disguised barrier easily. However, ethical considerations need to be taken into account since subjective barriers might restrict the PwDs’ autonomy, by restricting their privacy and causing them discomfort and boredom in situations where they require movement [44] . However, evidence is lacking to claim that subjective barriers prevent PwDs from wandering [44].

Another solution to keep PwDs from wandering is monitors and trackers that can be used to locate lost PwDs. This intervention has been met with controversy [45], and it has been argued that some of these measures restrict the PwD’s right to privacy [46]. Lastly, a more confining solution is the care village, such as “De Hogeweyk” near Amsterdam in the Netherlands, which is spacious yet contains only one inconspicuous main exit [47]. The concept of the care village is praised by some as an innovative solution [48, 49], yet there is also concern because the village itself can be seen as a form of “benevolent manipulation” [50].

To conclude, these alternative solutions pose similar problems to those of fake bus stops. In the following section, I will discuss how fake bus stops can still be a viable option to address the issue of wandering PwDs and assess whether or not they could provide an appropriate solution.

## Proposal and conclusion

To date there are no studies, which explored the benefits and risks of fake bus stops for PwDs. This limitation restricts the article’s discussion to experiences with alternative interventions, ethical arguments and practical experiences in regard to lying and deception. This also highlights the need for future research on the impact of fake bus stops.

Discussions with practitioners reveal that fake bus stops are very well received and PwD benefit from them. According to practitioners and caregivers, fake bus stops decrease the level of agitation and restlessness of PwD sitting at them [2].

Since fake bus stops in nursing homes resemble government issued bus stops in Germany, they have the potential to evoke memories PwD associate with real bus stops. These evoked memories could encourage social interactions among PwD, drawing on their personal experiences with bus stops. This could positively mitigate not only wandering, but also apathy in PwD.

Fake bus stops are intended to benefit PwD. Their purpose is to decrease wandering by giving PwD a place to go to and prevent them from walking on the streets. Some Practitioners argue that fake bus stops are effective in preventing PwDs from wandering and thus have the potential to enhance their well-being [4]. Moreover, lying and using deceptive techniques should only be done as a last resort [42]. The first choice should always be a non-deceptive method of care. Deception should never outweigh or replace truthfulness even when there are benevolent intentions motivating the deceptive act. The ethical standards of the medical profession should always be considered, e.g. the Declaration of Geneva by the World Medical Association [40] and the Hippocratic Oath. The Declaration of Geneva binds to prioritise the patient’s well-being and respect her or his autonomy. Respect for autonomy, is also one of the four key principles of biomedical ethics [51], and of great significance for patients in all fields of medical care. Therefore, people without dementia - whether caregivers, practitioners or relatives -, are obliged to examine closely and critically the purposes and intentions of the techniques that are used in interaction with PwD. This does not mean that deception in clinical routines is categorically inadmissible, but that one must carefully weigh the potential harm against the potential benefits. Ideally, PwD are to be included in the decision-making process to preserve their autonomy and freedom.

Arguably, the intention of building fake bus stop affects its permissibility. If the bus stop is intended to benefit PwD by alleviating psychological symptoms, then it is ethically permissible. If, however, the intention of fake bus stops is to make care less time consuming – which is very important for healthcare systems due to the lack of personnel – and to park challenging PwD at those stops, then building fake bus stops should be avoided and other options should be considered.

When considering the implementation of fake bus stops, care facilities should be aware of the following points:

1. Design: A less authentic design is less deceptive, and therefore less harmful to the PwD. when debunked. Additionally, a comfortable design is to be preferred, so that people without dementia may also enjoy sitting at the installations. For example, by adding bookshelves, comfortable sofas, and pictures, fake bus stops could be modified to recreational stations, encouraging social interaction and avoiding singling out PwD thus reducing the risk of PwD’s stigmatisation. Another way to limit PwD’s stigmatization is placing the fake bus stops in the common rooms rather than in the hallways or door entrances. Moreover, by modifying fake bus stops with pictures or screens with movies of landscapes, one could create a place where people are encouraged to share their travel experiences. This could create a place that stimulates interpersonal exchange not only between PwD, but among everyone who wants to get involved. This implementation would strengthen the exchange between PwD, relatives and staff.

2. Intention: A fake bus stops should only be implemented to enhance the well-being of PwD, not to replace interpersonal interactions. Thoughtful caregivers are needed to detect the psychic condition and needs of PwD when sitting at fake bus stops.

3. Potential harm: Although fake bus stops seem to have positive effects on well-being of PwD, caregivers should always be aware of the potential harm they can cause. As soon as a negative effect is noticed, nursing homes should abstain from this method.

## Abbreviations

BPSD: Behavioral and Psychological Symptoms in Dementia
PwD: Person with Dementia
